# Inoculant effects on limpograss silage nutritive value, fermentation characteristics, microbiology, and in vitro gas production

**DOI:** 10.1093/tas/txag125

**Published:** 2026-08-12

**Authors:** João V Lazarin, João M B Vendramini, Bruno I Cappellozza, Philipe Moriel, Hiran M S da Silva, André V de Miranda, Debora Siniscalchi

**Affiliations:** Texas A&M AgriLife Research and Extension Center, Stephenville, TX 76401, United States; Texas A&M AgriLife Research and Extension Center, Stephenville, TX 76401, United States; Novonesis, Lyngby, 2800, Denmark; IFAS | Range Cattle Research and Education Center, University of Florida, Ona, FL 33865, United States; IFAS | Range Cattle Research and Education Center, University of Florida, Ona, FL 33865, United States; Faculty of Land and Food Systems, The University of British Columbia, Vancouver, 248-2357, Canada; Department of Animal Science, Sao Paulo State University, Jaboticabal, SP, 14884-900, Brazil

**Keywords:** fermentation characteristics, limpograss, microbial inoculant, microbiome, nutritive value, silage

## Abstract

The objective of this study was to evaluate the effects of a microbial inoculant on nutritive value, fermentation parameters, microbiology, and gas production of ‘Gibtuck’ limpograss (*Hemarthria altissima* [Poir.] Stapf & C.E. Hubbard) silage. Treatments were silage treated or not with an inoculant containing a mixture of *Lactococcus lactis* and *Lentilactobacillus buchneri* (SiloSolve FC; Novonesis, Lyngby, Denmark) at 0.5 g/Mg of as-fed forage. Treatments were distributed in a randomized complete block design with 10 replicates (blocks), where each block consisted of one control and one inoculated mini silo. The experiment was conducted in Ona, FL, from March to July 2023. Twenty 9 m^2^ plots of established Gibtuck limpograss were harvested at 15-cm stubble height with 58 days of regrowth interval. Samples were chopped, treated, placed into mini-silos (capacity of 1.2 kg of fresh forage), and silos were sealed immediately. The silos were opened after 60 days for analysis. There was no difference (*P* ≥ 0.42) between the inoculant and control treatments on dry matter concentration, dry matter recovery, neutral detergent fiber (NDF), non-fiber carbohydrates, and total digestible nutrients concentrations. However, silage treated with inoculant had greater (*P* ≤ 0.04) crude protein, in vitro true dry matter digestibility, and NDF digestibility, and lesser acid detergent fiber concentrations than the control. The inoculant did not affect (*P* ≥ 0.28) silage pH and lactic acid concentrations but resulted in greater (*P* ≤ 0.02) acetic and propionic acids, and decreased butyric acid, isobutyric acid, and ammonia-N concentrations. Aerobic stability, and mold and yeast counts were not affected (*P* ≥ 0.28) by the inoculant treatment. At the genus level, there was no difference (*P* ≥ 0.54) in relative abundance of *Lentilactobacillus*, *Enterobacter*, *Enterococcus*, and *Weissella* among treatments, but silage inoculation tended to reduce *Clostridium* and *Pediococcus* (*P* ≤ 0.09), while increasing the relative abundance of *Lactococcus* and *Bacillus* (*P* ≤ 0.01). Limpograss silage treated with inoculant tended to reduce in vitro gas (*P* = 0.07) and methane production (*P* = 0.06) compared with the control. These results indicate that SiloSolve FC is an effective microbial inoculant for limpograss silage and suggest that it may potentially improve the quality of other warm-season perennial grass silages under subtropical and tropical conditions.

## Teaser

The use of SiloSolve FC improved the nutritive value and fermentation characteristics of limpograss silage, which resulted in greater nutrient preservation and reduced in vitro gas and methane production.

## Introduction

Limpograss is an important warm-season perennial forage species used in the southeastern United States (Wallau et al. 2016; Vendramini et al. 2021). Limpograss has been widely used due to its adaptability to poorly drained soils and greater digestibility at more advanced maturity stages compared to other warm-season perennial grasses (Quesenberry et al. 2018; Vendramini et al. 2021). Recently, the use of ‘Gibtuck’ limpograss, a hybrid cultivar developed by the University of Florida, has increased due to its greater herbage accumulation than previously released cultivars (Quesenberry et al. 2018).

The seasonal distribution of forage production in subtropical and tropical regions requires producers to conserve excess forage during periods of greater production to meet animal nutritional demands when forage availability is limited. Hay production is the predominant forage conservation method used in subtropical and tropical regions, but it is difficult because frequent rainfall and high humidity limit effective forage drying during periods of warm-season perennial grasses growth (Sollenberger et al. 2004). Prolonged field drying periods increase the risk of weather-related damage such as leaf loss, microbial spoilage, and leaching of soluble nutrients, which may reduce hay nutritive value (Collins 1995; Rotz 1995; Sollenberger et al. 2004). The thick stems of limpograss slow forage drying and often require mechanical conditioning to achieve safe baling moisture, increasing both the risk of forage nutritive value losses and operational costs (Quesenberry et al. 2004; Vendramini et al. 2021).

Silage is a practical alternative for conserving warm-season perennial grasses in subtropical and tropical regions, as it requires substantially less field drying period and forage processing (Arriola et al. 2015; Vendramini et al. 2016). However, warm-season perennial grasses have high moisture content and low water-soluble carbohydrate (WSC) concentrations (3–12% DM basis; Jensen et al. 2014; Weinert-Nelson et al. 2022), which limit pH decline and increase the likelihood of undesirable fermentation (Sollenberger et al. 2004; Vendramini et al. 2010).

Limited research has specifically evaluated limpograss silage. Vendramini et al. (2010) reported that Floralta limpograss had greater lactic acid concentration and lower pH than other warm-season grasses, indicating relatively improved fermentation; however, there was no inoculant effect on limpograss silage. More recently, Pereira Neto et al. (2024) demonstrated that limpograss silage can be used as a conserved forage for growing heifers, although animal performance was limited by low crude protein concentration, emphasizing the need to improve silage nutritive value.

Although adding microbial inoculants to silages is a commonly used practice to improve the quality of ensiled material (Adesogan et al. 2004; Cappellozza et al. 2025), responses in warm-season perennial grass have been inconsistent. Previous research has largely focused on other warm-season species, such as bermudagrass, with variable results depending on inoculant composition and forage characteristics (Vendramini et al. 2010, 2016; Gouvêa et al. 2020).

In addition to microbial inoculants, chemical or fermentable substrate additives have been used to improve the fermentation of tropical grasses (da Silva et al. 2026); however, their evaluation in limpograss silage systems remains limited. The available literature indicates that the effectiveness of additives in limpograss silage is not well established and requires further investigation (Vendramini et al. 2010).

SiloSolve FC (Novonesis, Lyngby, Denmark) is a commercial silage inoculant containing *Lactococcus lactis* and *Lentilactobacillus buchneri* that combines homofermentative and heterofermentative modes of action. This inoculant has demonstrated improvements in fermentation characteristics and aerobic stability in forage systems such as high-moisture corn and grass silages due to the complementary effects of rapid acidification and acetic acid production (Cappellozza et al. 2025). However, inoculant effectiveness is strongly influenced by forage chemical composition, epiphytic microbial populations, and management conditions, which vary widely among warm-season grasses and often result in inconsistent responses. To date, its effects have not been evaluated in limpograss silage, and therefore, its performance must be assessed within specific forage systems to determine suitability and improve the predictability of silage outcomes.

It is hypothesized that ensiling limpograss with SiloSolve FC improves nutrient preservation, fermentation characteristics, and aerobic stability, while reducing mold and yeast activity. The objective of this study was to evaluate the effects of adding SiloSolve FC on the nutritive value, fermentation characteristics, aerobic stability, yeast and mold populations, microbial relative abundance, and gas production of limpograss silage.

## Material and methods

The study was conducted at the University of Florida, Range Cattle Research and Education Center, Ona, FL (27°23′58.3″ N, 81°56′12.0″ W), from March to July 2023. The soil at the experimental area is classified as sandy siliceous, hyperthermic Alfic Alaquod (Eau Gallie sand), poorly drained with slow permeability (Vendramini et al. 2016). A Gibtuck limpograss hayfield was subdivided into 20 plots of 9 m^2^ with 1-m alleys between plots. The plots were staged in March 2023 at a stubble height of 7.5 cm and fertilized with 400 kg/ha of 20-05-20 (N-P-K) commercial fertilizer. The harvest was performed in May 2023 with a rotatory blade mower (Sensation Mow-Blo model 11F4-0; Sensation, Ralston, NE) set to the same stubble height (7.5 cm), and the forage was chopped to a particle size of approximately 14 ± 4 mm and dry matter (DM) at 28 ± 1.5%. The regrowth interval was targeted at 53 d.

Treatments consisted of a commercial microbial inoculant (SiloSolve FC, Novonesis, Lyngby, Denmark) and a control (no inoculant) arranged in a randomized complete block design with 10 replicates (blocks), where each block consisted of one control and one inoculated mini silo. The inoculant level was applied at 0.5 g/Mg of as-fed forage following manufacturer guidelines and consisted of a combination of *Lactococcus lactis* and *Lentilactobacillus buchneri* (68.1 × 10^9^ colony-forming units [CFU] per g of forage). The inoculant was diluted in 20 mL of deionized water, and the control treatment received the same volume of deionized water without inoculant. Inoculant and control treatments were sprayed over the forage material using a hand sprayer (Mainstays Spray Bottle, Walmart Inc., Bentonville, AR) while mixing the forage samples. Following application, forage samples were immediately packed into mini-silos (PVC pipes with rubber caps; 12.7-cm diameter × 26.7-cm height; 1.2 kg of fresh forage capacity). Packing density was approximately 162 ± 8 kg DM/m³. Silos were maintained for 60 days in a covered barn at ambient temperature and humidity. Silos were opened in July 2023.

### Laboratory analyses

Representative subsamples of pre-ensiled forage were dried at 60 °C for 48 h in a forced-air oven to determine the initial DM concentration. After silo opening, ensiled samples were dried at 60 °C for 48 h in a forced-air oven for DM determination and ground in a Wiley mill (Model 4, Thomas-Wiley Laboratory Mill, Thomas Scientific, Swedesboro, NJ) to pass a 1-mm screen. Ground samples were analyzed for crude protein (CP), neutral detergent fiber (NDF), acid detergent fiber (ADF), non-fiber carbohydrates (NFC), in vitro true digestibility (IVTD), NDF digestibility (NDFD), ammonia nitrogen (NH_3_-N), pH, lactic acid, volatile fatty acids (VFA) concentrations, and yeast and mold counts. Total digestible nutrients (TDN) were estimated according to Weiss et al. (1992).

Dry matter recovery was expressed as the proportional difference between the DM concentration before ensiling and the DM measured at silo opening and expressed as a percentage of the initial DM. Total nitrogen concentration was determined using the combustion method (AOAC 1999; model FP 2000; LECO Corporation, St. Joseph, MI), and CP was calculated as *N* × 6.25.

Neutral and acid detergent fiber concentrations were determined using fiber procedures described by Van Soest et al. (1991) and adapted for ANKOM 200 Fiber Analyzer (ANKOM Technology, Macedon, NY). Heat-stable α-amylase and sodium sulfite were included during NDF determination, and fiber values are reported inclusive of residual ash. Non-fiber carbohydrates were calculated by difference. In vitro true digestibility was determined using an ANKOM-adapted method (ANKOM 2005) based on Van Soest et al. (1966), with incubation in an ANKOM Daisy ^II^ Incubator. Neutral detergent fiber digestibility was subsequently calculated from the digestion residues using an ANKOM 200 Fiber Analyzer to calculate NDF digestibility.

Fermentation characteristics were evaluated using aqueous extracts prepared from fresh ensiled material diluted with deionized water and filtered before analysis (Vendramini et al. 2016). The extract pH was measured potentiometrically (Thermo Orion Posi-Phlo Electrode and Thermo Orion 410A meter; Orion Research Inc., Beverly, MA). Ammonia nitrogen concentration was determined using a colorimetric procedure described by Carlson (1978). Volatile fatty acid concentrations were quantified by gas chromatography following acidification and clarification of silage extracts (Vendramini et al. 2016). The silage extract was diluted 1:10, and yeast and mold populations were quantified on YM-11 agar (Neogen Corp., Lansing, MI; 6904A) following incubation at 32°C for 72 h (method 997.02; AOAC 1999).

Aerobic stability was evaluated using approximately 0.5 kg of silage per sample. The silage content was first removed from the mini silo, homogenized, and then a 0.5 kg subsample was transferred back into the mini silo. Each mini silo had one end sealed using a rubber cap secured with stainless steel clamps, while the opposite end was covered with a perforated plastic wrap to allow air exposure while minimizing excessive moisture loss. Temperature sensors (Onset Computer Corporation, Bourne, MA) were placed at the center of each mini-silo and programmed to record temperature every 30 min. The silos were maintained under controlled ambient conditions at 24 ± 0.5°C. Aerobic stability was expressed as the time (h) required for silage temperature to exceed ambient temperature by at least 2°C.

Microbial community composition was assessed using 16S rRNA gene sequencing. All silage samples (*n* = 20; one per experimental unit) were analyzed for microbial relative abundance following procedures described by Lazarin et al. (2026). Briefly, total DNA was extracted from samples using the PowerFood Microbial DNA Isolation Kit (MO BIO Laboratories, Inc., Carlsbad, CA) according to the manufacturer instructions. The V4 region of the bacterial and archaeal 16S rRNA gene was amplified by PCR using barcoded universal primers (515F/806R) compatible with the Illumina MiSeq platform (Caporaso et al. 2012). Amplicons were purified, normalized, pooled in equimolar concentrations, and sequenced using paired-end chemistry (2 × 301 bp) on an Illumina MiSeq system (Illumina Inc., San Diego, CA). Sequencing and initial bioinformatic processing were conducted by a commercial laboratory (FERA Laboratory, College Station, TX) using Illumina Local Run Manager Metagenomics workflow (version 2.0.0). Taxonomic classification was performed using the Greengenes database (version 13.5). Sequence data were processed to determine the microbial relative abundance at the genus level. Microbial relative abundance was expressed as percentages. Detailed sequencing depth metrics and specific quality-filtering parameters were not provided in the original report and were handled within the default settings of the Illumina Local Run Manager pipeline.

In-vitro gas production was measured using an automated ANKOM RF Gas Production System (ANKOM, Macedon, NY). Briefly, 10 independent 250-mL sealed bottles (corresponding to 10 experimental units; 5 replicates per treatment across 2 treatments) equipped with an automated pressure and temperature sensor were used as gas-collection modules. Sensors recorded cumulative gas production every minute. Each gas collection bottle contained 18 g of ensiled material collected immediately after silo opening. Rumen fluid was collected from a rumen-cannulated steer (950 kg BW), filtered through four layers of cheesecloth, and immediately transported to the laboratory in a prewarmed and sealed container. The canulated steer was fed ad libitum stargrass (*Cynodon nlemfuesis* Vanderyst) hay along with daily supplementation of 1.0 kg of soybean meal and 1.0 kg of cottonseed meal. Upon arrival at the laboratory, rumen fluid was mixed with McDougall’s buffer at a 4:1 ratio (McDougall 1948), and 100 ml of the mixture was added per module. Bottles were maintained under controlled ambient conditions at 24 ± 0.5°C for 336 h. Although pressure data were recorded at 1-min intervals, gas production data were summarized into 24-h intervals (from 24 to 336 h), resulting in 14 time points for statistical analysis. Gas samples were collected using 1-L Tedlar bags (Supelco Analytical, Bellefonte, PA) connected from the gas-collection modules. Methane (CH_4_) concentration was determined using a 7890 B gas chromatograph (Agilent Technologies Inc., Santa Clara, CA) equipped with a flame ionization detector for CH_4_ determination. The gas and CH_4_ production were expressed on a digestible DM basis.

### Statistical analysis

Statistical analyses were performed using the MIXED procedure of SAS (SAS Institute Inc., Cary, NC). Experimental units were mini silos. The experiment was conducted in a randomized complete block design, with each of the 10 replicates representing a block containing one control and one inoculated mini silo. Response variables related to nutritive value, fermentation characteristics, yeast and mold counts, aerobic stability, and microbial relative abundance were analyzed by fitting mixed-effects models. Treatment (control vs. inoculant) was included in the model as a fixed effect, and block (replicate) was included as a random effect to account for variability among silos. Residual error represented variability among experimental units within blocks. This design was selected to account for variability with the sequential preparation of silos, as the ensiling process occurred over time, and conditions could vary slightly between preparation periods. Blocking allowed treatments to be compared under similar conditions, thereby reducing experimental error and improving the precision of treatment effects.

Alpha diversity was assessed using the Shannon index and compared between treatments using a Wilcoxon rank-sum test due to non-normal data distribution. Beta diversity was evaluated using Bray–Curtis dissimilarity and visualized by principal coordinates analysis (PCoA), with differences in community structure tested by PERMANOVA.

For gas production, a subset of experimental units (5 blocks per treatment; total *n* = 10 mini silos) was used. Treatment, time of incubation, and their interaction (treatment × time) were considered fixed effects, and block (replicates) was included as a random effect.

The statistical model for gas production was:


$$Y_{\left(\mathrm{ijk}\right)}=\mu +T_{i}+B_{j}+{\mathrm{Time}}_{k}+{\left(T\times \mathrm{Time}\right)}_{ik}+\varepsilon_{\left(ijk\right)},$$

where T_i_ is the fixed effect of treatment, B_j_ is the random effect of block (replicate), Time_k_ is the repeated effect of incubation time, and ε_(ijk)_ is the residual error.

Time of incubation was analyzed as repeated measurements, and multiple covariance structures (including compound symmetry and autoregressive) were evaluated, with compound symmetry selected based on the least Akaike information criterion value. Statistical significance was declared at *P* ≤ 0.05 and tendencies at 0.05 < *P* ≤ 0.10. Treatments and interaction effects that were not significant (*P* > 0.10) are not presented in the Discussion section. The means reported were least-squares means, and pairwise comparisons were conducted using the PDIFF statement of SAS (SAS Institute Inc., Cary, NC).

## Results

The inoculant did not affect limpograss silage pH and lactic acid concentration (*P* ≥ 0.28) but resulted in greater acetic acid, propionic acid, and decreased butyric acid, isobutyric acid, and NH_3_-N concentrations (*P* ≤ 0.02; Table 1). The inoculant treatment tended to increase total acid concentrations compared with the control (*P* = 0.06; Table 1).

**Table 1:** Fermentation characteristics of limpograss (*Hemarthria altissima* [Poir.] Stapf & C.E. Hubbard) silage treated with SiloSolve FC (0.5 g/Mg of as-fed forage; Novonesis, Lyngby, Denmark) inoculant containing a combination of *Lactococcus lactis* and *Lentilactobacillus buchneri* (68.1 × 10^9^ CFU/g) and control (no inoculant; 10 samples per treatment)^1^.

	Treatment			
Item	Control	Inoculant	SE	*P*-value
**DM recovery, % initial DM**	96.0	96.0	1.40	0.88
**pH**	4.2	4.2	0.04	0.33
**Lactic acid, % of DM**	1.6	1.4	0.15	0.28
**Acetic acid, % of DM**	2.0	2.5	0.13	0.02
**Propionic acid, % of DM**	1.0	1.6	0.10	<0.01
**Butyric acid, % of DM**	0.2	0.0	0.03	<0.01
**Isobutyric acid, % of DM**	0.4	0.1	0.04	<0.01
**Total acids, % of DM**	5.0	5.6	0.25	0.06
**Ammonia-N, % of N**	5.3	4.4	0.19	<0.01
**Mold count, log CFU/g of silage as fed**	1.1	1.1	0.11	0.91
**Yeast count, log CFU/g of silage as fed**	2.5	2.1	0.49	0.55
**Aerobic stability, hours**	137	143	10	0.28

1 Limpograss silage samples were collected 60 d after ensiling.

Aerobic stability, and mold and yeast populations in limpograss silage were not affected by the inoculant treatment (*P* ≥ 0.28; Table 1).

There was no difference between the inoculant and control treatments on DM concentration and recovery, NDF, TDN, and NFC concentrations (*P* ≥ 0.42); however, limpograss silage treated with inoculant had greater CP, IVTD, and NDFD and lesser ADF concentrations than the control (*P* ≤ 0.04; Table 2).

**Table 2:** Nutritive value of limpograss (*Hemarthria altissima* [Poir.] Stapf & C.E. Hubbard) silage treated with SiloSolve FC (0.5 g/Mg of as-fed forage; Novonesis, Lyngby, Denmark) inoculant containing a combination of *Lactococcus lactis* and *Lentilactobacillus buchneri* (68.1 × 10^9^ CFU/g) and control (no inoculant; 10 samples per treatment)^1^.

	Treatment			
Item^2^	Control	Inoculant	SE	*P*-value
**DM, % of silage**	25.7	25.6	0.49	0.85
**CP, % of DM**	8.0	8.5	0.20	0.04
**ADF, % of DM**	40.1	38.7	0.47	0.02
**NDF, % of DM**	67.0	66.9	0.58	0.89
**TDN^3^, % of DM**	54.7	54.9	0.17	0.42
**NFC, % of DM**	14.1	13.8	0.49	0.65
**NDFD, % of DM**	51.0	53.0	0.50	0.02
**IVTD, % of DM**	54.0	56.0	0.50	0.01

1 Limpograss silage samples were collected 60 d after ensiling.

2 DM, dry matter; CP, crude protein; ADF, acid detergent fiber; NDF, neutral detergent fiber; TDN, total digestible nutrients; NFC, non-fiber carbohydrates; NDFD, neutral detergent fiber digestibility; IVTD, in vitro dry matter digestibility.

3 Calculated as described by Weiss et al. (1992).

**Figure 1:**
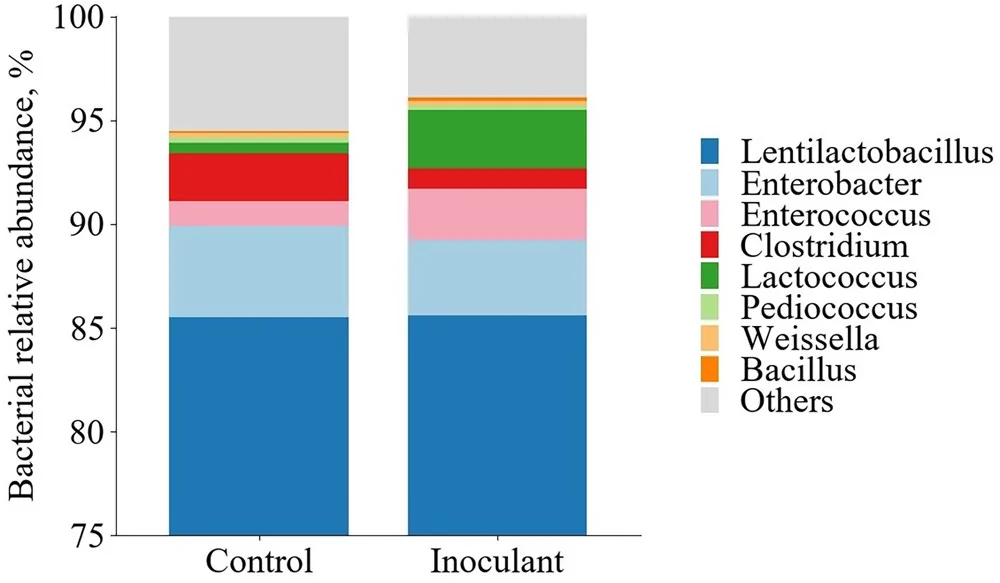
Bacterial relative abundance by genus of limpograss (*Hemarthria altissima*) silage treated with SiloSolve FC (0.5 g/Mg of as-fed forage; Novonesis, Lyngby, Denmark) inoculant containing a combination of *Lactococcus lactis* and *Lentilactobacillus buchneri* (68.1 × 10^9^ CFU/g) and control (no inoculant). Relative abundance of *Lentilactobacillus*, *Enterobacter*, *Enterococcus*, *Weissella*, and *Others* (mean = 85, 4.0, 1.8, 0.2, and 4.7%, respectively) did not differ among treatments (*P* > 0.05). Relative abundance of *Clostridium* and *Pediococcus* tended to lesser in the treated silage than the control (1.0 vs. 2.3%, *P* = 0.07; 0.21 vs. 0.29%, *P* = 0.09, respectively). Relative abundance of *Lactococcus* and *Bacillus* was greater in the treated silage than the control (2.8 vs. 0.5%, *P* < 0.05; 0.2 vs. 0.1%, *P* < 0.05, respectively).

**Figure 2:**
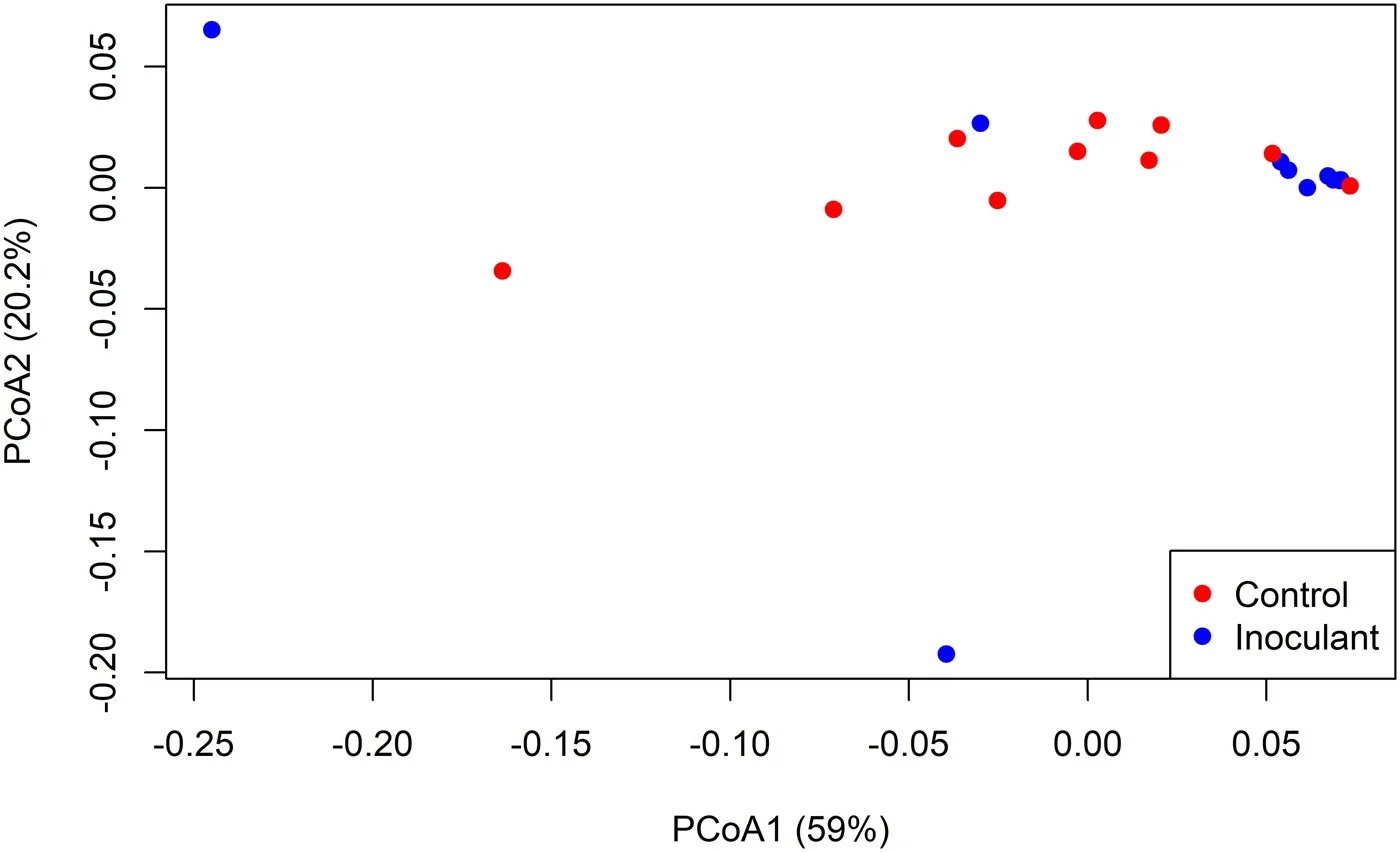
Principal coordinates analysis (PCoA) of Bray–Curtis dissimilarity showing beta diversity of bacterial communities at the genus level in limpograss (*Hemarthria altissima*) silage treated with SiloSolve FC (0.5 g/Mg of as-fed forage; Novonesis, Lyngby, Denmark) containing *Lactococcus lactis* and *Lentilactobacillus buchneri* and control silage (no inoculant). Each point represents one silage sample (*n* = 20; 10 samples per treatment). Community structure did not differ between treatments according to PERMANOVA (R² = 0.058; *P* = 0.297).

The microbiome data were analyzed at the genus level. There was no difference in the relative abundance of *Lactobacillus*, *Enterobacter*, *Enterococcus*, *Weissella*, and others (mean = 85, 4.0, 1.8, 0.2, and 4.7%, respectively) among treatments (*P* ≥ 0.54; Fig. 1). However, limpograss silage treated with inoculant tended to decrease the relative abundance of *Clostridium* and *Pediococcus* compared with the control (1.0 vs. 2.3%, *P* = 0.07; 0.21 vs. 0.29%, *P* = 0.09, respectively; Fig. 1). Inoculated limpograss silage had greater relative abundance of *Lactococcus* and *Bacillus* (2.8 vs. 0.5%, *P* < 0.01; 0.2 vs. 0.1%, *P* = 0.01, respectively; Fig. 1) than the control. Shannon diversity index ranged from 0.36 to 1.50 across samples (*n* = 20), with no differences observed between treatments (*P* = 0.39). Beta diversity analysis showed substantial overlap between treatments in PCoA ordination (Fig. 2), and PERMANOVA confirmed no differences in microbial community structure (R² = 0.058, *P* = 0.30).

At the species level, the inoculant was effective in increasing the relative abundance of *Lactococcus lactis* from 0.02 to 0.48% (*P* = 0.002) and *Lentilactobacillus buchneri* from 1.4 to 1.9% (*P* = 0.03).

**Figure 3:**
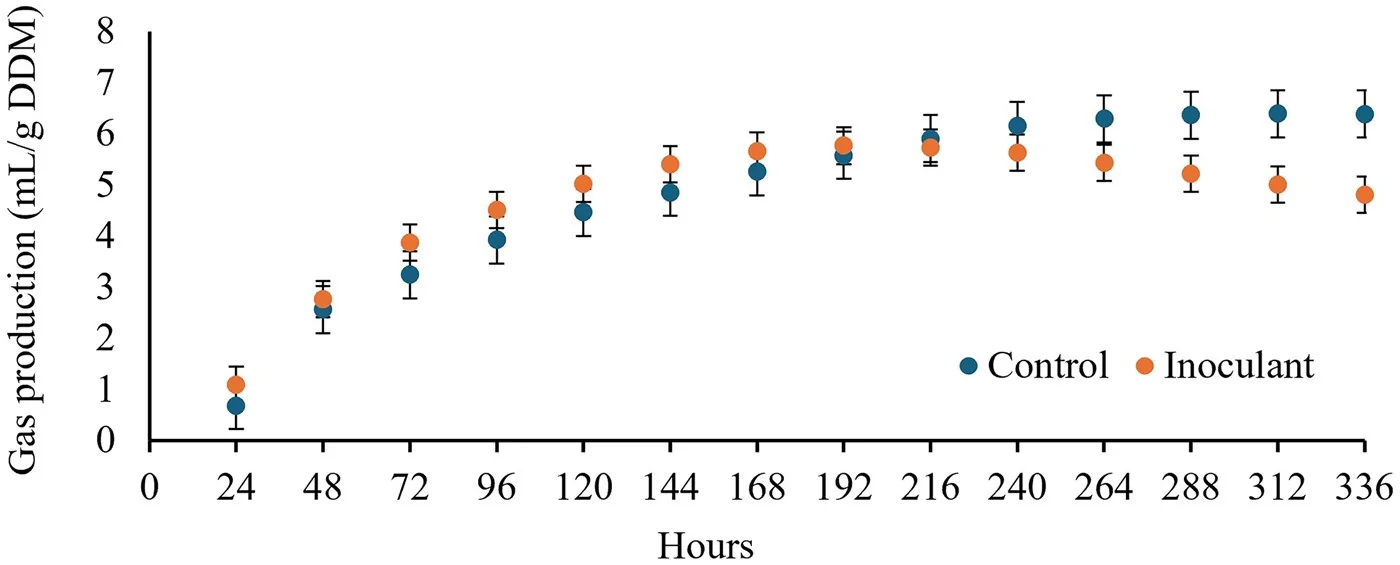
In-vitro gas production per gram of digestible dry matter of limpograss (*Hemarthria altissima*) silage treated with SiloSolve FC (0.5 g/Mg of as-fed forage; Novonesis, Lyngby, Denmark) inoculant containing a combination of *Lactococcus lactis* and *Lentilactobacillus buchneri* (68.1 × 10^9^ CFU/g) and control (no inoculant) across time (0 to 336 hours).

The effects of treatment × incubation time tended to be detected (*P* = 0.07) for silage gas production. The gas production in limpograss silage did not differ between the inoculant treatment and control from 24 to 264 hours; however, silage treated with inoculant had less gas production from 288 to 336 hours post-ensiling (Fig. 3). Additionally, limpograss silage treated with inoculant tended to decrease in vitro methane production (mL/g DDM) compared with the control (0.011 vs. 0.014 mL/g DDM; *P* = 0.06).

## Discussion

### Fermentation characteristics

Inoculated limpograss silage had a greater acetic acid concentration than the control, indicating that heterolactic fermentation was predominant during ensiling. The fermentation pathways of warm-season grasses are typically characterized by an acetate-dominated heterolactic pathway because of their low WSC concentrations (Bates et al. 1989; Dean et al. 2005). Additionally, the *Lentilactobacillus buchneri* likely used early lactic acid production from *Lactococcus lactis* to increase acetic acid and potentially 1,2-propanediol production by converting lactic acid during late fermentation (Muck et al. 2018). Although *Lentilactobacillus buchneri* does not directly produce propionic acid, this metabolic pathway may contribute to the formation of intermediates (1,2-propanediol) that can be further catabolized to propionic acid by other bacteria present in the silage (Oude Elferink et al. 2001; Krooneman et al. 2002), which likely justifies the greater propionic acid production for the inoculant treatment compared to the control. Conversely, previous studies using a range of homo- and heterofermentative bacterial inoculants did not observe changes in acetic and propionic acid concentrations after inoculation, which was associated with limited WSC concentrations (Vendramini et al. 2010; Gouvêa et al. 2020).

Limpograss silage treated with inoculant had less concentrations of butyric acid, isobutyric acid, and NH_3_-N compared with control silage. Butyric acid should ideally be absent in well-preserved silage, as its presence indicates clostridial fermentation (Castle and Watson 1985), with concentrations above 0.5% of DM generally considered indicative of poor silage quality (Kung 2001; Muck 2010). Although the butyric acid level in the control treatment was below (0.2% of the DM) the acceptable limit, the inoculant successfully reduced butyric acid concentrations. Clostridial activity in silage is characterized by the production of butyric acid from sugar and lactic acid, extensive proteolysis resulting in elevated NH_3_-N concentration, and the formation of isobutyric acid from amino acid deamination (McDonald et al. 1991). Therefore, the reductions in butyric acid, isobutyric acid, and NH_3_-N concentrations in inoculated silage are consistent with the reduced clostridial activity. Clostridial presence in the inoculated treatment was likely decreased due to unfavorable proliferation conditions (Oude Elferink et al. 2001; Cappellozza et al. 2025). Total acid concentration tended to increase in inoculated limpograss silage compared with the control, likely reflecting the greater acetic and propionic acid concentrations.

The lack of inoculant effect on DM recovery in this study is consistent with previous findings in warm-season perennial grass silages. Vendramini et al. (2016) reported that microbial inoculants, including formulations containing *Lentilactobacillus buchneri*, did not affect DM recovery of bermudagrass silage and that DM concentration at ensiling was the primary driver of DM losses. High-DM silage had a lesser WSC concentration than the control, likely due to greater loss of starch, fructosans, sugars, and other readily available carbohydrates through respiration (Moser 1980). Bermudagrass ensiled at approximately 52% DM achieved nearly complete DM recovery; conversely, silage ensiled at lower DM concentrations (∼22%) had reduced DM recovery to 96%, likely due to greater release of CO_2_ and water (Pitt 1990).

Given the relatively low pre-ensiling DM concentration in the present study (mean = 28 ± 1.5%%), both treatments likely experienced some CO_2_ and water losses; however, the observed DM recovery (96%) indicates that these losses were minimal and that overall preservation was efficient despite the relatively low DM concentration at ensiling (Pitt 1990). Nonetheless, the lower DM concentration in both inoculated and control treatments may explain the similar DM recovery observed. Additionally, the absence of differences in mold and yeast counts between treatments suggests that both silages had similar levels of aerobic spoilage organisms, which can contribute to DM losses during storage and feed-out.

It was expected that greater acetic acid and propionic acid concentrations in the inoculated silage would reduce mold and yeast counts due to their antifungal properties (Weinberg and Muck 1996; Oude Elferink et al. 2001; Muck et al. 2018; Jatkauskas et al. 2024; Kok et al. 2024); however, there was no difference in mold and yeast counts among treatments. The lack of acetic acid and propionic acid effects on yeast and mold were likely due to limited yeast and mold counts in the control treatment, which was also observed by da Silva et al. (2026). Corroborating with these results, there was no difference in aerobic stability among treatments. The lack of treatment effects on yeast and mold counts likely explains the similar aerobic stability among treatments, given that fungal populations are associated with improved silage stability when silage is exposed to air (Kung et al. 2018). Conversely, Jatkauskas et al. (2024), using the same inoculant in corn silage, reported improved aerobic stability associated with reduced yeast and mold populations following inoculation, likely reflecting the substantially greater baseline mold and yeast observed in the control group (9.22 and 9.02 log CFU/g, respectively). Additionally, Arriola et al. (2021) observed that heterofermentative bacterial inoculants can prevent yeast proliferation and improve aerobic stability as early as 7 to 15 d of silage storage; however, more pronounced effects are observed after prolonged fermentation (90 d and longer).

### Nutritive value

Neutral detergent fiber concentration was not affected by the inoculant, whereas ADF concentration was greater in the control. This suggests that differences in fermentation dynamics between treatments influenced the structure and accessibility of cell wall components during ensiling (Jiang et al. 2020). This interpretation is supported by the greater IVTD and NDFD observed in inoculated silage, indicating increased accessibility of fiber fractions. Hemicellulose represents a substantial proportion of NDF; therefore, minor changes in cellulose have proportionally less effect on NDF concentration than on ADF. Similarly, da Silva et al. (2026) observed that the use of propionic acid alone or in combination with a microbial inoculant containing homo- and heterofermentative bacteria reduced ADF concentrations while NDF concentrations did not change in bermudagrass silage.

Consistent with these responses, increases in NDFD and IVTD were observed in the inoculated silage. These responses suggest that the reduction in ADF concentrations due to inoculation affected silage digestibility. Vendramini et al. (2016) observed that adding molasses to bermudagrass silage decreased NDF and ADF concentrations, increasing NDFD and IVTD concentrations; however, microbial inoculant did not affect these parameters. Kang et al. (2009) evaluated the effects of inoculating whole-plant corn silage with an inoculant containing *Lentilactobacillus casei* and *buchneri*, reporting greater NDF and in situ DM digestibility for the treated silage compared to the untreated silage. The *Lentilactobacillus buchneri* strain produces ferulic acid esterase, which cleaves ferulate cross-links in cell-wall arabinoxylans, potentially enhancing fiber digestion directly or increasing cell-wall subsequent cellulolytic or xylanatic hydrolysis (Kang et al. 2009). Enhanced cellulolytic activity may have increased the accessibility of cellulose to microbial enzymes, thereby improving fiber digestibility without increasing NFC concentration or total energy density. The NFC is a calculated fraction largely driven by NDF concentration and the absence of treatment effects on NDF explains the lack of an inoculant response in NFC (Weiss et al. 1992). Consequently, improvements in NDFD and IVTD may be the result of greater utilization of fiber fraction rather than shifts in nutrient partitioning, which explains the lack of change in TDN (Oba and Allen 1999). Additionally, TDN results were estimated, and the associated prediction error may have affected the potential to detect treatment differences (Weiss et al. 1992).

Greater CP concentration observed in inoculated limpograss silage compared with the control is associated with lower NH_3_-N concentration in the inoculant treatment. Reduced NH_3_-N indicates decreased proteolysis during ensiling and improved nitrogen preservation, a response commonly associated with suppression of clostridial activity (McDonald et al. 1991; Kung et al. 2018). This interpretation is supported by the lesser relative abundance of *Clostridium* observed in the inoculated silage, as *Clostridium* spp. are primarily responsible for extensive proteolysis and amino acid deamination, leading to NH_3_-N accumulation (Pahlow et al. 2003; Muck et al. 2018). da Silva et al. (2026) reported that a reduction in clostridial presence decreased proteolytic activity and resulted in greater CP preservation.

### Microbial relative abundance

The reduction in the relative abundance of *Clostridium* in treated silages indicates that the greater acetic and propionic acid concentrations indirectly suppressed clostridial proliferation. The combination of homo- and heterofermentative inoculants promotes anaerobic conversion of lactic acid to acetic acid and 1,2-propanediol, creating an environment less favorable for *Clostridia*, which rely on lactic acid and neutral conditions to proliferate (Oude Elferink et al. 2001; Cappellozza et al. 2025). da Silva et al. (2026) reported that the addition of propionic acid along with a microbial inoculant (Early Sile Advance, Micron Bio-Systems Inc., Bridgewater, U.K.; *P. acidilactici*, *P. pentosaceus*, *L. plantarum*, and *L. brevis*) reduced the relative abundance of *Clostridium*. The reduction of this genus is beneficial because *Clostridium* is associated with undesirable silage fermentation and negative animal health impact (Kung et al. 2018). Additionally, clostridial fermentation is associated with reduced feed intake (Grant and Ferraretto 2018).

The reduced relative abundance of *Pediococcus* and increased abundance of *Lactococcus* suggest that the inoculant containing *Lactococcus Lactis* may have selectively suppressed some lactic acid bacteria, such as *Pediococcus*, while favoring others, such as *Lactococcus*. Corroborating these results, at the species level, the relative abundance of *Lactococcus Lactis* and *Lentilactobacillus buchneri* increased in the inoculated treatment, likely due to inoculant bacterial species composition (Jalpolsaen et al. 2022).

Among the predominant bacterial genera in silage, *Lentilactobacillus* is typically the most abundant (Jalpolsaen et al. 2022; da Silva et al. 2026), which is consistent with the present results. Although no treatment differences were detected, *Lentilactobacillus* accounted for an average of 85% of the relative microbial abundance across treatments. In addition, the relative abundance of other bacterial genera did not differ among treatments, indicating that treatment effects occurred primarily within key functional groups associated with silage fermentation.

Despite these differences, alpha diversity (Shannon index) did not differ between treatments, and beta diversity analysis indicated no changes in microbial community structure. The principal coordinates analysis ordination showed substantial overlap between treatments, and PERMANOVA confirmed the absence of treatment effects on community structure.

These findings indicate that the observed shifts in specific genera represent targeted compositional changes within an otherwise stable, Lentilactobacillus-dominated microbiome rather than a broad restructuring of the silage microbial community. Importantly, the reduction in the relative abundance of *Clostridium* despite the absence of changes in alpha and beta diversity suggests that the inoculant selectively suppressed undesirable taxa without altering overall community structure. This targeted response further supports that the microbial community remained stable while undergoing functionally beneficial shifts. Sequencing depth and read-quality metrics were not available from the sequencing provider; therefore, microbial diversity results should be interpreted primarily as relative compositional differences among treatments.

### Gas production

Despite no differences in aerobic stability, inoculated limpograss silage produced less gas between 288 and 336 h of incubation and tended to reduce methane emissions. This suggests that prolonged air exposure (>288 h), beyond the period captured by aerobic stability measurements, likely promoted yeast activity in the control silage, contributing to increased gas production. Following air exposure, yeasts proliferate rapidly and metabolize residual sugars, producing carbon dioxide (Kung et al. 2018). Although yeast and mold counts and aerobic stability (measured up to 143 h) did not differ between treatments in the present study, the immediate silo closure after harvest likely limited early microbial differences. In contrast, Jatkauskas et al. (2024) reported that delaying ensiling by 24 h increased yeast and mold counts and reduced aerobic stability, supporting the effects of extended oxygen exposure in stimulating yeast-driven deterioration.

The tendency for lower CH_4_ production may be explained by differences in silage fermentation characteristics and substrate availability. Methane formation in anaerobic systems occurs via the reduction of CO_2_ with hydrogen (CO_2_ + 4H_2_ → CH_4_ + 2H_2_O), and thus depends on the availability of reducing equivalents (H_2_) (Moss et al. 2000; Hristov et al. 2013). In the present study, inoculated silage had greater concentrations of propionic acid, which is associated with metabolic pathways that utilize reducing equivalents (glucose + 4[H] → 2 propionate + 2H_2_O). Although these pathways are primarily described in rumen fermentation systems, the higher concentration of propionate in the inoculated silage may reflect a shift in fermentation end-products toward propionate that reduces the availability of fermentable substrates and limits H_2_ generation during in vitro incubation. Consequently, less H_2_ may have been available for methanogenesis, resulting in lower CH_4_ production.

However, it should be noted that methane production in this study was evaluated using an in vitro system, and these mechanisms are inferred rather than directly measured. In addition, previous studies using various microbial inoculants, including *Lentilactobacillus buchneri* and *Lactococcus lactis*, have reported no effects on total gas or CH_4_ production, indicating that responses can vary depending on experimental conditions and silage characteristics (Dunière et al. 2015; Ellis et al. 2016).

## Conclusion

Application of a microbial inoculant containing *Lactococcus lactis* and *Lentilactobacillus buchneri* improved the nutritive value and fermentation characteristics of limpograss silage produced under subtropical conditions compared to the non-treated silage, resulting in better nutrient preservation. Inoculated silage had greater CP concentration and reduced NH_3_-N and undesirable fermentation products, indicating improved nitrogen conservation and suppression of clostridial activity.

Inoculated silage also produced less cumulative gas during in vitro incubation and tended to reduce methane production.

These results indicate that SiloSolve FC is an effective inoculant for improving fermentation quality and preserving nutrients in limpograss silage and may be beneficial in other warm-season perennial grass silage systems. Additional research is needed to confirm its effectiveness across forage species.

## List of abbreviations

ADF: Acid detergent fiber
CFU: Colony-forming units
CP: Crude protein
DM: Dry matter
IVTD: In vitro true digestibility
NDF: Neutral detergent fiber
NDFD: Neutral detergent fiber digestibility
NFC: Non-fiber carbohydrates
NH_3_-N: Ammonia-nitrogen
PCoA: Principal coordinates analysis
TDN: Total digestible nutrients
WSC: Water-soluble carbohydrates
